# Oncolytic vesicular stomatitis virus alone or in combination with JAK inhibitors is effective against ovarian cancer

**DOI:** 10.1016/j.omton.2024.200826

**Published:** 2024-06-08

**Authors:** Karen Geoffroy, Victor Mullins-Dansereau, Kim Leclerc-Desaulniers, Mélissa Viens, Marie-Claude Bourgeois-Daigneault

**Affiliations:** 1 Cancer and Immunopathology Axes, CHUM Research Centre, Montreal, QC H2X 0A9, Canada; 2 Institut du cancer de Montréal, Montreal, QC H2X 0A9, Canada; 3 Department of Microbiology, Infectious Diseases and Immunology, Faculty of Medicine, University of Montreal, Montreal, QC H3C 3J7, Canada

**Keywords:** ovarian cancer, oncolytic virus, VSV, JAK inhibitors, ruxolitinib

## Abstract

Therapy-resistant ovarian cancers have a poor prognosis and novel effective treatment options are urgently needed. In this study, we evaluated the therapeutic efficacy of the oncolytic vesicular stomatitis virus (VSV) against a panel of patient-derived ovarian cancer cell lines of all epithelial subtypes. Notably, we found that most of the cell lines were sensitive to VSV virotherapy. With the objective of improving treatment efficacy for the oncolytic virus-resistant cell lines, we tested various combinations with ovarian cancer standard of care drugs: olaparib, carboplatin, paclitaxel, doxorubicin, cyclophosphamide, and gemcitabine. While none of these combinations revealed to be beneficial, further experiments demonstrated that the antiviral interferon pathway was functional in VSV-resistant cell lines. Given that interferons signal through Janus kinase (JAK)-STAT to mediate their antiviral function, we tested combinations of oncolytic VSV with clinically relevant JAK inhibitors. Our results show that combining VSV with various JAK inhibitors, including ruxolitinib, enhances VSV virotherapy and treatment efficacy. Altogether, we show that VSV, either as a stand-alone treatment or in combination with JAK inhibitors provides an effective therapeutic option for ovarian cancer patients.

## Graphical abstract

**Figure:**
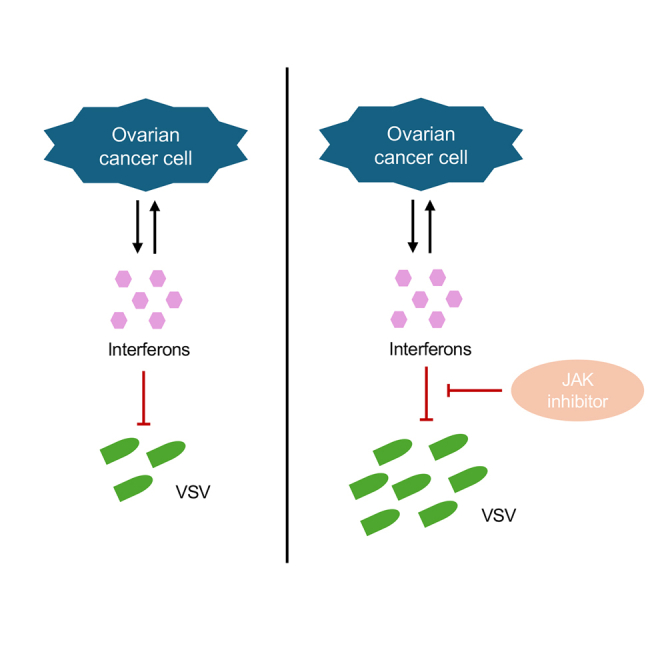

## Introduction

With a mortality rate of 65% in 2022, ovarian cancer is the deadliest gynecological cancer and the fifth most lethal cancer among women in the United States.^1^ Standard of care therapies include combinations of taxane (paclitaxel) and platinum (carboplatin) chemotherapeutic agents administered either in a neo-adjuvant setting or after a debulking surgery.^2^ Given the high recurrence rate, as well as the frequent evolution of chemoresistance, second-line treatments are often needed. These include doxorubicin (a topoisomerase II inhibitor), cyclophosphamide (an alkylating agent), gemcitabine (a pyrimidin analog), and olaparib (a poly ADP ribose polymerase [PARP] inhibitor), among others.^3^^,^^4^ Despite these many options, patients often relapse, thus underscoring the urgent need for additional effective treatments for ovarian cancer patients.

Immunotherapeutic agents such as cancer-killing oncolytic viruses (OVs) are powerful tools against cancer.^5^ OVs can selectively replicate in tumor cells in part due to defects acquired during malignant transformation.^6^ A promising OV is the vesicular stomatitis virus (VSV), more specifically VSVΔ51, an oncolytic variant of VSV with an engineered mutation in its matrix (M) protein^7^ that renders the virus sensitive to antiviral pathways that are often defective in cancer.^8^ VSV is a multi-mechanistic anticancer agent that directly infects and kills cancer cells, destroys the tumor vasculature,^9^ induces apoptosis,^10^ attracts immune cells within the tumor microenvironment,^11^ and triggers anti-tumor immunity.^12^

In this study, we sought to evaluate the potential of VSV against ovarian cancer. Using a panel of 33 patient-derived ovarian cancer cell lines and select xenograft models, we tested oncolytic VSV both as a stand-alone treatment, as well as in combination with various anticancer drugs. We found the majority (21/33) of the cell lines tested were sensitive to VSV virotherapy. As with other cancer therapeutics, not all neoplasms are sensitive to OV therapy. Since various drugs have been shown to synergize with OVs and improve the efficacy of the treatment in virus-resistant cancer models,^13^^,^^14^ we investigated whether ovarian cancer standard of care drugs could enhance VSV therapy; however, combinations with olaparib, carboplatin, paclitaxel, doxorubicin, cyclophosphamide, and gemcitabine failed at enhancing VSV.

The ovarian cancer tumor microenvironment is known to contain antiviral interferons (IFNs), which have both pro- and anti-tumor functions^15^ and we found all virus-resistant ovarian cancer cell lines tested to be IFN competent. OVs have previously been combined with Janus kinase (JAK) inhibitors,^16^^,^^17^ which block IFN signaling, therefore leaving the cell defenseless against infection. To date, 11 JAK inhibitors are approved by the Food and Drug Administration (FDA) for various indications, including ruxolitinib and baricitinib, which are JAK1/2 inhibitors,^18^^,^^19^ and fedratinib, a selective JAK2 inhibitor.^20^ While none of these drugs are currently used for ovarian cancer treatment, ruxolitinib is currently undergoing clinical testing in this patient population in combination with paclitaxel and carboplatin (NCT02713386). Therefore, we aimed to combine VSV with these three compounds and found the combinations to be very effective at overcoming virus resistance. Our data show that combining VSV with JAK inhibitors enhances VSV both *in vitro* and *ex vivo*, also improving treatment efficacy in human ovarian cancer xenografts. Taken together, we show that VSV, either alone or administered in combination with clinically relevant JAK inhibitors, could be effective as a therapeutic option for ovarian cancer patients. Given the clinical status of the JAK inhibitors tested, our approach has the potential to be rapidly translated to the clinic.

## Results

### Patient-derived ovarian cancer cell lines are sensitive to oncolytic VSV

**Figure 1:**
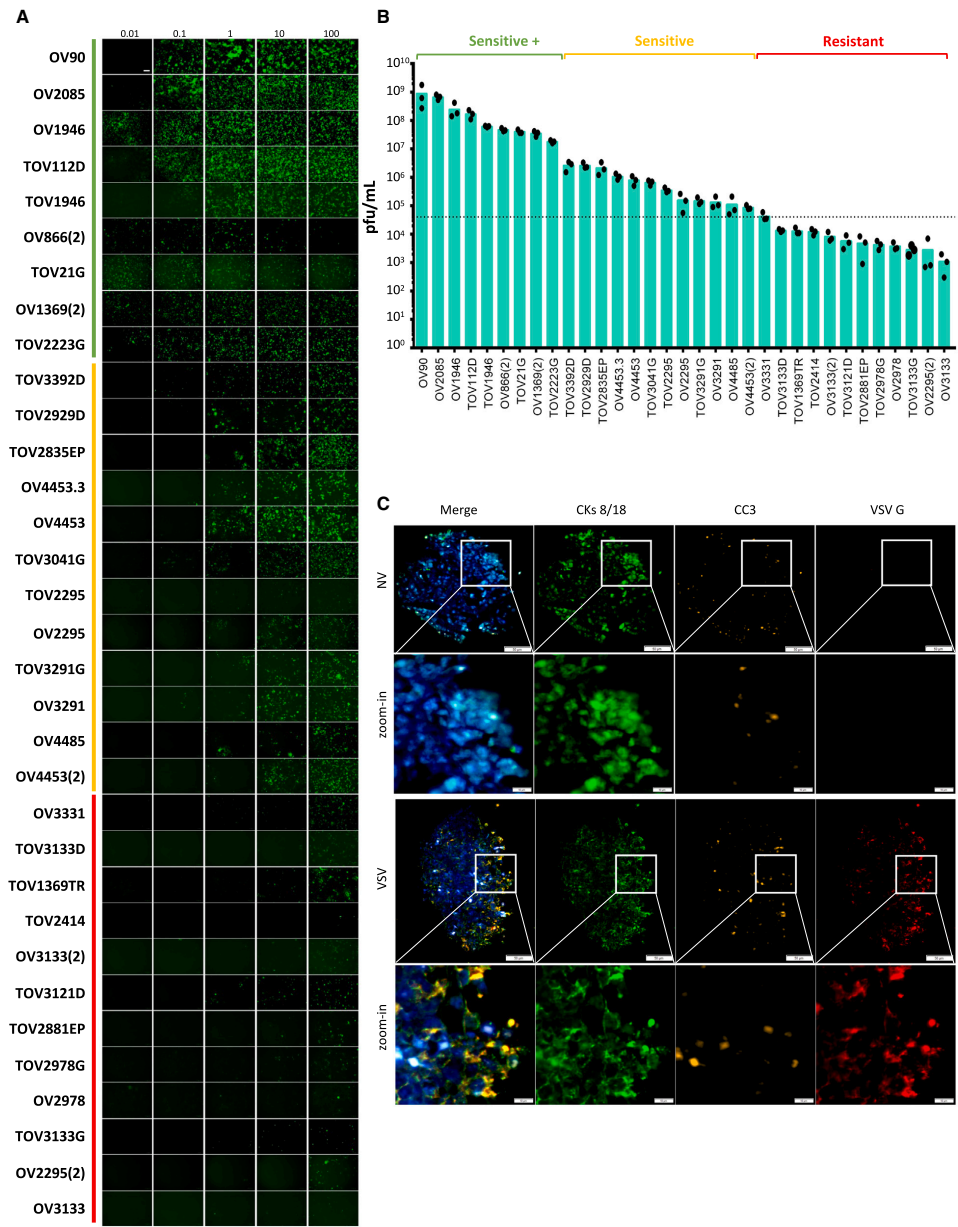
Ovarian cancer cells are sensitive to oncolytic VSV infection (A) Fluorescence imaging of ovarian cancer cell lines infected with VSVΔ51-YFP at the indicated MOIs for 24 h. Scale bar, 100 μm. (B) Viral outputs from cells in (A) infected at an MOI of 0.1 as measured by plaque assays (*n* = 3). The dotted line indicates virus input. (C) Representative fluorescence microscopy images of TOV112D xenografts infected *ex vivo* with 1.5 × 10^6^ PFU/mL of VSVΔ51-YFP for 24 h. The images show nucleus in blue (DAPI), VSV G in red, cleaved caspase-3 (CC3) in yellow, and epithelial (tumor) cells (CKs 8/18) in green. NV, no virus. Whole core scale bar, 50 μm, close-up scale bar, 10 μm.

To evaluate the therapeutic potential of oncolytic VSV against ovarian cancer, we tested infection in a panel of 33 human epithelial ovarian cancer cell lines of various subtypes (described in Table 1). To do so, we used VSVΔ51-YFP and infection was assessed by fluorescence imaging and plaque assays 24 h post-infection. Strikingly, we found that most cell lines were sensitive to VSV with fluorescent cells detected at low MOIs (0.1 and 0.01) and infectious virus particles being produced upon infection (Figures 1A, 1B, and S1A). We then classified the cell lines according to their viral sensitivities based on the viral outputs produced upon infection at an MOI of 0.1 (Figure 1B). Cell lines for which viral output was at least 3 logs higher than input 24 h post-infection were considered very sensitive (sensitive+, 27.3% of the cell lines), the ones that produced virus but for which the output was less than 3 logs more than input were classified as sensitive (36.4% of the cell lines), and the ones for which viral output was equal or lower than input were classified as resistant. In line with these findings, we found virus-sensitive and -sensitive+ cells to be less viable after 24 h of infection at an MOI of 10 compared with virus-resistant cell lines (36.4% of the cell lines) (Figure S1B).

**Table 1:** Characteristics of the ovarian cancer cell lines used in this study

Cell line	Cancer type	Stage	Age at diagnosis	Treatment	VSV sensitivity
OV90	Adenocarcinoma	IIIC	64	Naive	Sensitive +
OV2085	HGSC	IIIC	63	PTX/CBP	Sensitive +
OV1946	HGSC	IIIC	75	Naive	Sensitive +
TOV112D	Endometrioid carcinoma	IIIC	42	Naive	Sensitive +
TOV1946	HGSC	IIIC	75	Naive	Sensitive +
OV866(2)	HGSC	IIIC	60	PTX/CBP	Sensitive +
TOV21G	Clear cell carcinoma	III	62	Naive	Sensitive +
OV1369(2)	HGSC	IIIC	58	PTX/CBP Dox, Top	Sensitive +
TOV2223G	HGSC	IIIC	89	Naive	Sensitive +
TOV3392D	Clear cell carcinoma	IIIC	42	5-FU, epirubicine, CPP, trastuzumab	Sensitive
TOV2929D	HGSC	IIIC	77	Naive	Sensitive
TOV2835EP	HGSC	IIIC	62	PTX/CBP	Sensitive
OV4453.3	HGSC	IIIC	70	Naive	Sensitive
OV4453	HGSC	IIIC	70	Naive	Sensitive
TOV3041G	HGSC	IVA	61	PTX/CBP	Sensitive
TOV2295	HGSC	IIIC	59	Cisplatin/Top PTX/CBP, Dox	Sensitive
OV2295	HGSC	IIIC	59	Naive	Sensitive
TOV3291G	HGSC	IIIC	59	Naive	Sensitive
OV3291	HGSC	IIIC	59	Naive	Sensitive
OV4485	HGSC	IIIC	55	PTX/CBP	Sensitive
OV4453(2)	HGSC	IIIC	70	Naive	Sensitive
OV3331	Adenocarcinoma	IIIC	72	PTX/CBP Epothilone B	Resistant
TOV3133D	HGSC	IIIC	52	Naive	Resistant
TOV1369TR	HGSC	IIIC	58	Naive	Resistant
TOV2414	Mucinous carcinoma	IIIC	63	Naive	Resistant
OV3133(2)	HGSC	IIIC	52	PTX/CBP, Dox CBP/Gemcitabine	Resistant
TOV3121D	HGSC	IIIC	62	PTX/CBP	Resistant
TOV2881EP	HGSC	IIIC	56	PTX/CBP, Tamoxifen	Resistant
TOV2978G	HGSC	IIIC	63	Naive	Resistant
OV2978	HGSC	IIIC	63	Naive	Resistant
TOV3133G	HGSC	IIIC	52	Naive	Resistant
OV2295(2)	HGSC	IIIC	59	Cisplatin/Top PTX/CBP, Dox	Resistant
OV3133	HGSC	IIIC	52	Cisplatin/Top PTX/CBP, Dox	Resistant

Name, cancer type, stage at the time of surgery, age at diagnosis, and oncolytic VSV sensitivities determined from results found in Figure 1 of ovarian cancer cell lines used in this study. Nomenclature: cell lines derived from primary tumors (TOV), ascites (OV), left ovary (G), and right ovary (D). Stages of ovarian cancer follow the FIGO classification. HGSC, high-grade serous carcinoma; PTX, paclitaxel; CBP, carboplatin; Dox, doxorubicin; Top, topotecan; 5-FU, 5-fluorouracil; CPP, cyclophosphamide. Oncolytic viruses represent a promising strategy against cancer; however, not all cancers respond to the treatment. Here, Bourgeois-Daigneault and colleagues show, using a collection of human cell lines, as well as xenografts, that VSV is a promising virotherapy against ovarian cancer as a stand-alone therapy or combined with JAK inhibitors.

The infectibility of a subset of cell lines selected to be representative of each viral sensitivity category was further assessed using tumor xenografts *ex vivo*. TOV112D, OV866(2), TOV3041G, and OV3331 tumors were micro-dissected and infected with VSVΔ51-YFP for 24 h. Live fluorescence microscopy pictures show that all samples were infected by the virus (Figure S1C). We designed an immunofluorescence (IF) panel to visualize tumor cells (anti-cytokeratins [CKs] 8/18), infection (anti-VSV G), and cell death (anti-cleaved caspase 3 [CC3]). We found that infection was indeed observed in *ex vivo* infected xenografts as more VSV-positive cells were observed (Figure 1C). Given that all VSV-infected cells were also CKs 8/18+, our data show that infection was restricted to tumor cells. Increased cell death (CC3+) was also observed in infected samples. Taken together, our results show potential for the use of oncolytic VSV against ovarian cancer.

### Ovarian cancer standard of care drugs do not improve VSV virotherapy

**Figure 2:**
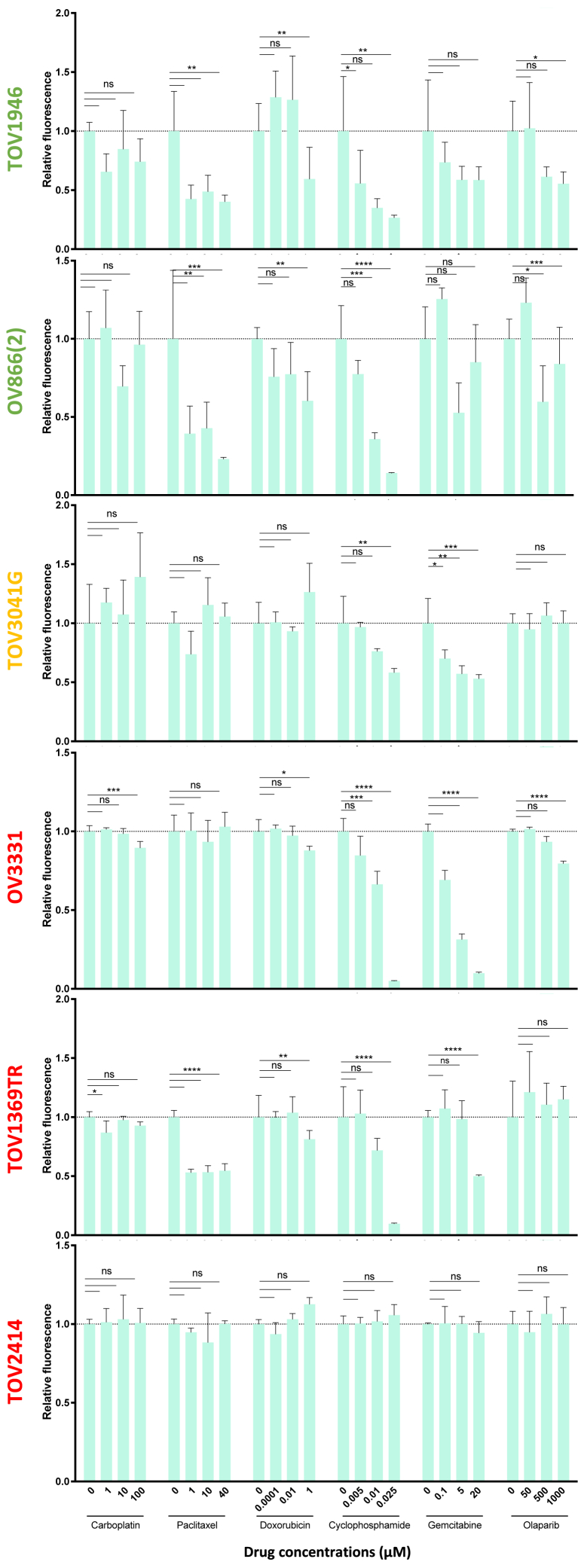
Combinations of VSV with various anticancer drugs do not enhance VSV Relative fluorescence signal (infection) of ovarian cancer cell lines infected with VSVΔ51-YFP 4 h post-carboplatin, -paclitaxel, -doxorubicin, -cyclophosphamide, -gemcitabine, or -olaparib treatment at the indicated concentrations compared with no drug controls. MOIs used: 0.01 for TOV1946 and OV866(2), 1 for TOV3041G and OV3331 and 10 for TOV1369TR and TOV2414. The fluorescence was measured 24 h post-infection using an Ensight multimode plate reader. Two-way ANOVA test (*n* = 3), ns: *p* > 0.05, ∗*p* ≤ 0.05, ∗∗*p* ≤ 0.01, ∗∗∗*p* ≤ 0.001, ∗∗∗∗*p* ≤ 0.0001.

While over 60% of our ovarian cancer cell lines were sensitive to VSV therapy, 36% were found to be resistant (Figure 1B). With the objective of enhancing the viral sensitivity of the resistant cell lines, we tested combinations with various clinically relevant drugs that are currently used for ovarian cancer treatment (paclitaxel, carboplatin, doxorubicin, cyclophosphamide, gemcitabine, or olaparib). Although some drug candidates such as paclitaxel, doxorubicin, and olaparib were expected to enhance viral infection based on previous reports by our group and others showing enhancement of VSV,^14^ or from other studies evaluating adenovirus^21^ and herpes simplex virus (HSV),^22^ respectively, none of the combinations tested succeeded at enhancing VSV replication (Figures 2 and S2) or cancer cell death (Figure S3) in the six ovarian cancer cell lines tested. Therefore, the various virus-drug combinations tested are not promising strategies to treat ovarian cancer, at least in our tumor models.

### Ovarian cancer cells are IFN competent

**Figure 3:**
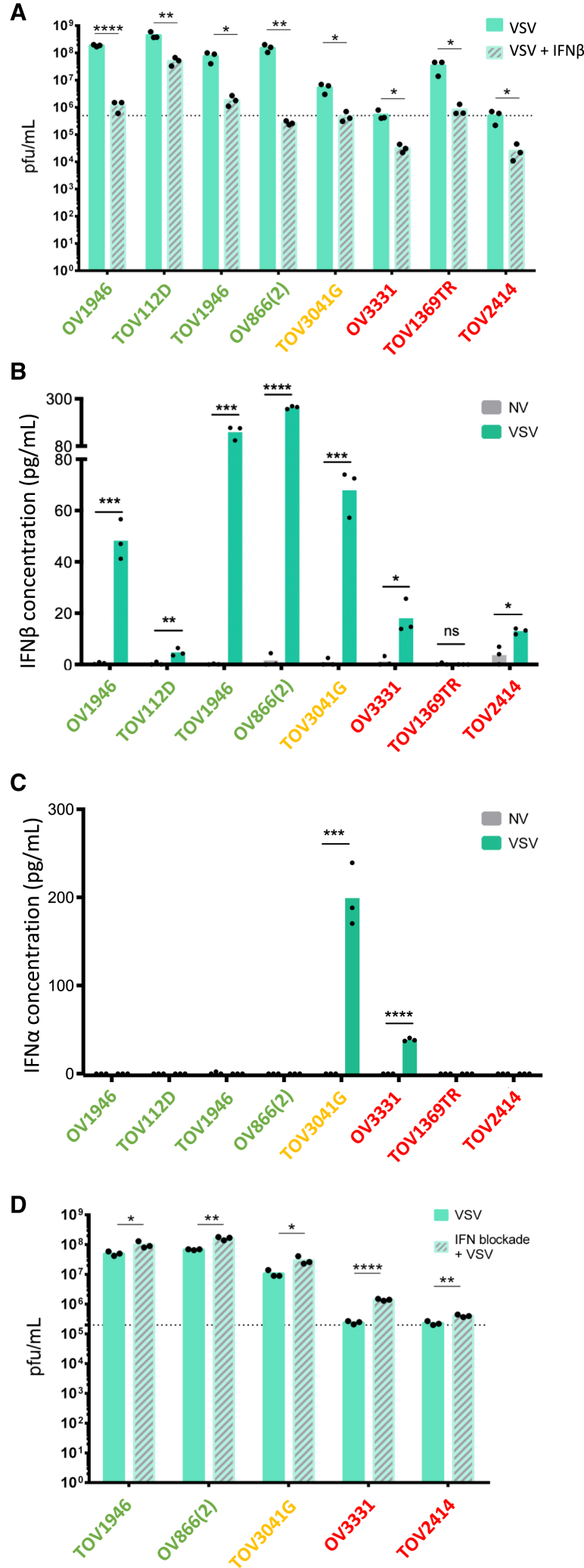
Ovarian cancer cell lines are IFN competent (A) Viral outputs from ovarian cancer cell lines pre-treated with human IFNβ for 4 h prior to infection with VSVΔ51-YFP at an MOI of 10 for 24 h were measured by plaque assay. (B) IFNβ and (C) IFNα production from the same samples as in (A) were quantified by ELISA. (D) Viral outputs from ovarian cancer cells treated with type I IFN neutralizing antibodies and infected 4 h later with VSVΔ51-YFP at an MOI of 10. Dotted lines represent viral inputs. Multiple t test (*n* = 3), ns: *p* > 0.05, ∗*p* ≤ 0.05, ∗∗*p* ≤ 0.01, ∗∗∗*p* ≤ 0.001, ∗∗∗∗*p* ≤ 0.0001.

With the objective of enhancing VSV therapy in virus-resistant ovarian cancer cells, we sought to uncover the biological mechanism underlying virus resistance. IFNs are important antiviral cytokines and even though cancer cells often have defects in the pathway (either production or response^8^), some remain IFN competent, which confers resistance to viral infection. To determine if our cells were IFN competent, we first tested their infectibility post-IFN treatment to assess their responsiveness. We selected eight cell lines representative of each viral sensitivity category and found all of them to be protected from viral infection by IFNβ pre-treatment, therefore demonstrating their responsiveness to the antiviral cytokine (Figures 3A and S4A). We next assessed IFN production by ELISA and found that all cell lines, except for TOV1369TR, produced IFNβ upon infection (Figure 3B) and OV3331 and TOV3041G also produced IFNα (Figure 3C). These data suggest that blocking IFNs could enhance virus infection in most ovarian cancer cell lines. To test this, we used a mixture of blocking antibodies targeting both the type I IFN receptor, as well as several type I IFNs. As expected, IFN blockade resulted in increased virus production in all tested cell lines (Figures 3D and S4B). Taken together, our data show that blocking type I IFNs enhances oncolytic VSV replication in ovarian cancer.

### JAK inhibitors synergize with oncolytic VSV

**Figure 4:**
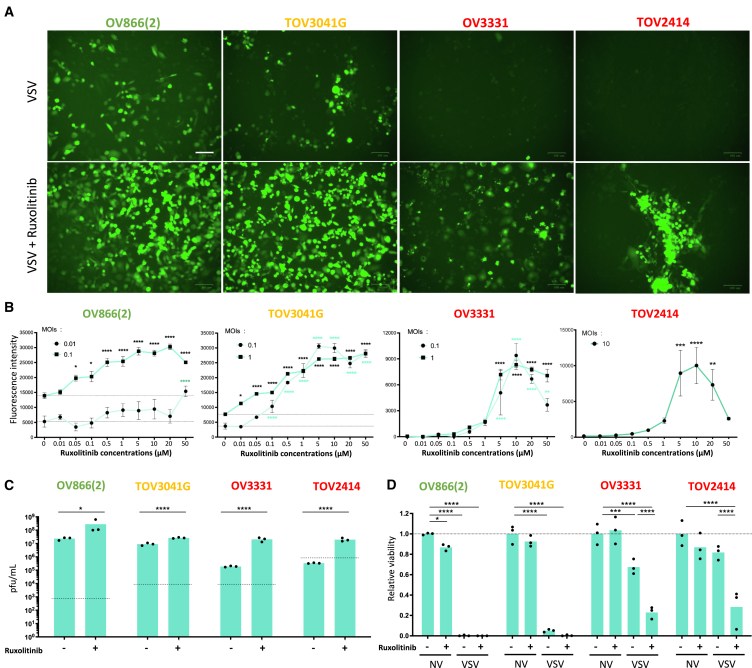
Ruxolitinib increases VSV replication and cancer cell death (A) Fluorescence imaging pictures of ovarian cancer cell lines treated with ruxolitinib (10 μM) for 4 h prior to infection with VSVΔ51-YFP at various MOIs (0.01 for OV866(2), 0.1 for TOV3041G, 1 for OV3331, and 10 for TOV2414). Scale bar, 100 μm. (B) Quantification of the fluorescent signal from cells pre-treated with a range of ruxolitinib concentrations 4 h prior to infection with VSVΔ51-YFP at the indicated MOIs. (C) Viral replication and (D) Coomassie blue stain from cells infected with VSVΔ51-YFP and treated with ruxolitinib (5 μM) after 48 h post-infection were measured. Two-way ANOVA test (*n* = 3), ∗*p* ≤ 0.05, ∗∗*p* ≤ 0.01, ∗∗∗*p* ≤ 0.001, ∗∗∗∗*p* ≤ 0.0001. Dotted lines indicate the fluorescent signal detected in non-treated control conditions (B), viral inputs (C), or viability of non-infected and non-treated control conditions (D).

**Figure 5:**
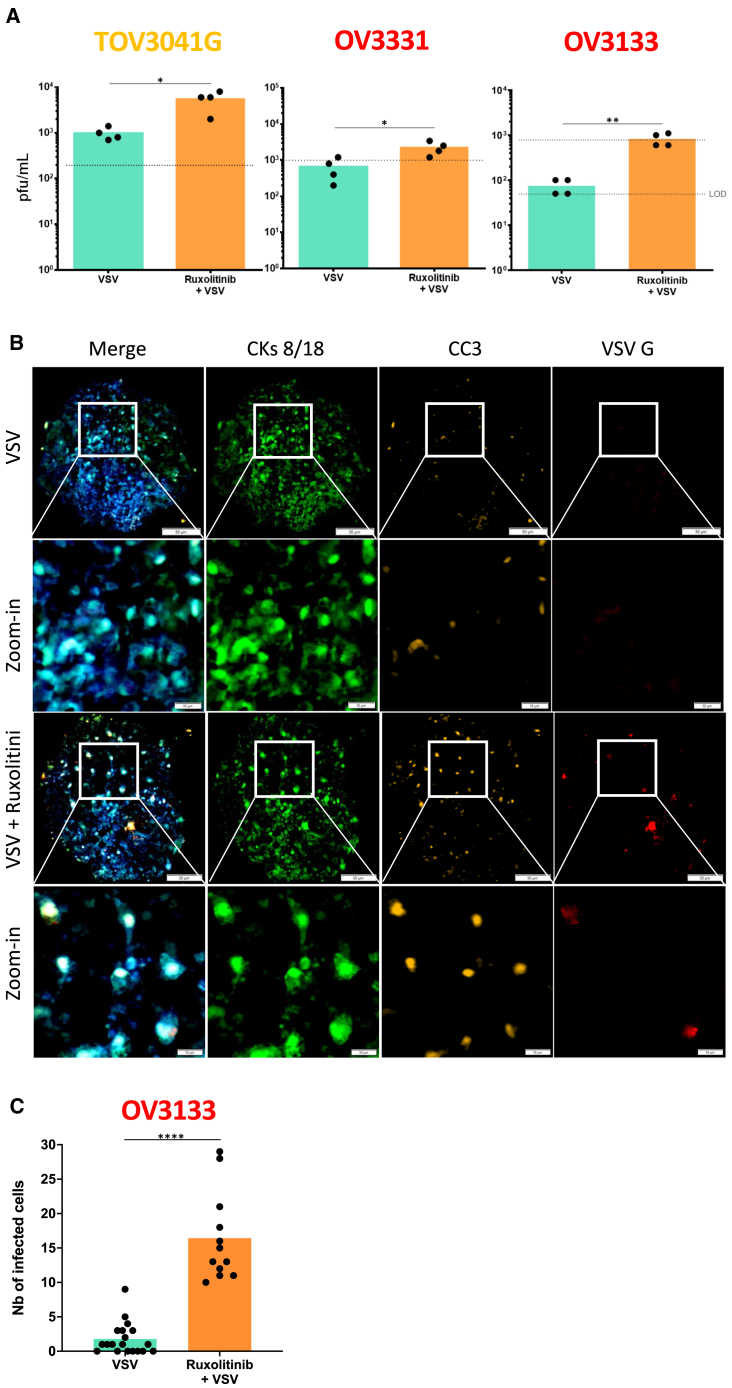
Combination of VSV and ruxolitinib led to better viral replication *ex vivo* (A) Virus titers from TOV3041G, OV3331, and OV3133 xenografts pre-treated with ruxolitinib (5 μM) for 4 h prior to infection with VSVΔ51-YFP (1.5 × 10^6^ PFU/mL). Virus outputs were measured 24 h post-infection by plaque assay (*n* = 4). (B) Representative IF images of OV3133 samples stained for nuclei (DAPI in blue), VSV G (red), apoptotic cells (CC3 in yellow), and epithelial tumor cells (CKs 8/18 in green). Scale bar, 50 μm, close-up scale bar, 10 μm. (C) Number of positive cells for VSV per sample were counted manually (*n* ≥ 12). Multiple t test, ∗*p* ≤ 0.05, ∗∗*p* ≤ 0.01, ∗∗∗∗*p* ≤ 0.0001.

To exploit IFN blockade to enhance oncolytic VSV using clinically relevant drugs, we opted to target IFN signaling. We tested combinations with ruxolitinib, baricitinib, and fedratinib, which are three FDA-approved drugs that prevent IFN signaling through JAK inhibition. As expected, combinations with all three drugs improved viral infection 24 h post-infection (Figures 4A, 4B, S5A–S5C, S6A, and S6B). We also observed increased virus production when the cells were infected following ruxolitinib or baricitinib treatment (Figures 4C and S6C). Impressively, quantification of tumor cell viability 48 h post-infection revealed that even VSV-resistant cell lines (OV3331 and TOV2414) were almost completely killed when co-treated with JAK inhibitors (Figures 4D and S6D). Interestingly, while combining VSV with fedratinib enhanced viral infection based on yellow fluorescent protein (YFP) transgene expression (Figure S6B), it did not affect virus production. Also, it increased cancer cell death in the viral-resistant cell lines (Figures S6E and S6F) but to a lesser extent than the two other JAK inhibitors tested. To validate that JAK inhibition could potentiate VSV virotherapy in tumors, we performed *ex vivo* infections with ruxolitinib pre-treatment and found that, as expected, the drug enhanced VSV production in TOV3041G, OV3331, and OV3133 xenografts *ex vivo* experiment (Figure 5A). OV3133 samples were stained for VSV and CC3, and both markers seemed to be increased in the treatment combination samples (Figure 5B). A quantification of the number of infected cells per image revealed that more infected cells were indeed found in tumor cores from ruxolitinib-treated tumors (Figure 5C).

**Figure 6:**
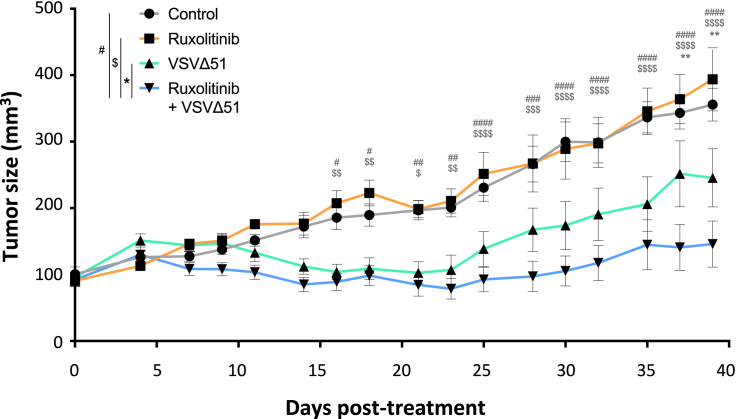
Combination of VSV and ruxolitinib enhances VSV efficacy Tumor growth curves of OV866(2) xenograft-bearing mice treated with ruxolitinib (5 μM) or vehicle (PBS) i.p. 4 h before intratumoral injection of VSV (10^e^8 PFU) or PBS. Control (*n* = 5), ruxolitinib (*n* = 4), VSVΔ51 (*n* = 8), and ruxolitinib+VSVΔ51 (*n* = 8). Multiple t test, ∗*p* ≤ 0.05, ∗∗*p* ≤ 0.01, ∗∗∗*p* ≤ 0.001, ∗∗∗∗*p* ≤ 0.0001.

We next tested the efficacy of the combination *in vivo*. To do so, we selected a xenograft model (OV866(2)) that is highly sensitive to VSV *in vitro*, for which viral enhancement by ruxolitinib was confirmed *in vitro* and that allowed for the consistent growth of tumors in immunocompromised mice. Upon reaching a volume of 90 mm^3^ mice received either vehicle or ruxolitinib 4 h prior to intratumoral injection of oncolytic VSV or PBS. Concentrations of the various drugs were determined based on previous studies.^16^^,^^23^ We found that VSV administration allowed for better tumor control as early as day 16 post-treatment compared with non-infected mice (Figure 6). In addition, the combination led to even better tumor control and showed a significant difference with VSV administered alone starting at day 37. While mice from the ruxolitinib-treated (one mouse) and VSV-treated (two mice) cohorts had already reached endpoint (Figure S7A), the remaining animals were euthanized at day 53. Tumors were extracted and weighed. Although the biggest tumors were not part of the analysis because the animals had already been killed, the pictures and tumor weights show that both groups receiving oncolytic VSV had smaller tumors compared with the control (mean of 177.6g) and ruxolitinib (mean of 225.2g) groups, with the smallest tumors in the combination group (mean of 119.9 g vs. 115.8 g for VSV alone) (Figures S7B and S7C). Taken together, our results support the combination of VSV with JAK inhibitors as a promising strategy against ovarian cancer.

## Discussion

In this study, we evaluated the efficacy of oncolytic VSV as an anticancer agent against ovarian cancer and found that VSV infects and kills over 60% of ovarian cancer cells and that its combination with JAK inhibitors further improves therapy.

As observed with other cancer treatments, the response to VSV virotherapy is heterogeneous and a subset of human ovarian cancer cell lines (30%) were resistant to infection. Despite the reported defects in IFN production and responsiveness found in many cancers,^8^ all but one of our ovarian cancer cell lines tested were IFN competent. In line with this, we found that blocking IFNs resulted in improved VSV infection (Figure 3D). Although IFN defects contribute to the specificity of OVs against cancers,^24^ other pathways, such as PI3K/Akt, Myc, Notch, RTK-RAS, p53, and b-catenin/Wnt^25^ can also be dysregulated and contribute to virus susceptibility. Nonetheless, we did not observe any sign of toxicity, which is in line with reports by others that have shown the safety of combining JAK inhibitors with OVs.^17^^,^^26^

Many studies have reported synergistic OV-drug combinations. A promising combination that was found to be effective by our group and others is the combination of the anticancer drug paclitaxel and virotherapies, including oncolytic VSV.^14^^,^^27^^,^^28^ Unfortunately, this combination, as well as combinations with other ovarian cancer standards of care: carboplatin, doxorubicin, cyclophosphamide, gemcitabine, and olaparib all failed at improving VSV infection in ovarian cancer cell lines (Figure 3). As expected, some ovarian cancer cell lines were sensitive to the various anticancer drugs, which resulted in cell death and reduced viral replication. For cell lines that are highly sensitive to the drugs such as TOV1946 with paclitaxel, the use of a low concentration (0.2 μM) was sufficient to cause cell death, which did not allow for the testing of the combinations at concentrations that are clinically relevant. Notably, cancer cell death, which is the goal of the treatment, was never found to be reduced with the different combinations compared with either treatment alone. Due to the high sensitivity of some cell lines to the drugs alone, synergistic effects were difficult to achieve, which resulted in a decreased fluorescent signal observed in these conditions. In addition, some cell lines were intrinsically resistant to the drugs tested. For example, four cell lines were found to be resistant to paclitaxel, which can happen as a result of increased drug efflux, glutathione detoxification, or hyperactivation of the PI3K/Akt pathway,^29^ thereby having no impact on the tumor cell.

Because type I IFNs are key players in the first line of defense against many viruses and VSV is known to be highly sensitive to IFNβ,^30^ we first investigated the IFN competence (responsiveness and production) of selected human ovarian cancer cell lines (Figures 3A and 3B). Given that cancer cells are often reported to be IFN defective,^8^ the finding that all our tested cell lines were IFN competent was rather surprising. Other biological mechanisms are known to contribute to viral resistance or sensitivity. For example, high baseline expression of IFN-stimulated genes,^31^^,^^32^ hypoxia,^33^ as well as altered metabolism^34^ and epigenetic modifications^35^ are known to alter virus infection. While hypoxia is likely not contributing to viral resistance in our culture conditions, the other mechanisms could be playing a role in our system. In addition, given that viruses benefit from rapid cell division,^36^^,^^37^ it is reasonable to speculate that slow-growing cells, such as TOV1369TR,^38^ would be more resistant to virus infection.

We tested combinations with JAK inhibitors, which interfere with IFN signaling. More particularly, JAK1 acts with tyrosine kinase 2 downstream of the type I IFN receptor to trigger STAT1/2 activation. For type II IFN, JAK1 and JAK2 activate STAT1. Of the three JAK inhibitors investigated, ruxolitinib and baricitinib both inhibit JAK1 and JAK2, while fedratinib only inhibits JAK2. Interestingly, we found that both ruxolitinib and baricitinib, but not fedratinib, improved virus-mediated cancer cell death and virus production (Figures 4, S5, and S6), with the best results observed with 5 μM of ruxolitinib combined with high MOIs. This suggests that blocking JAK1 (type I IFNs) is more effective than inhibiting type II IFN, at least *in vitro* where IFNγ is absent. Given that immune cells such as T cells, natural killer cells, and macrophages are the main sources of IFNγ,^39^ inhibiting type II IFN might still be efficient *in vivo*.^40^ Importantly, the treatment was well tolerated and the mice did not show any sign of toxicity, pain, or neurotoxicity. In line with our observations, other studies have already assessed the safety of combining VSV and ruxolitinib in mice and did not observe differences in mouse weights post-treatment.^16^^,^^17^^,^^26^

Our experiments in tumor xenografts (Figures 5 and 6) confirmed the VSV enhancement effect of ruxolitinib and support the enhanced treatment efficacy with the virus-drug combination in virus-resistant ovarian cancers. Moreover, considering that ruxolitinib-mediated inhibition of IFN signaling is largely reduced 24 h post-administration,^41^ it is reasonable to think that the combination will be safe in humans hence confirming the potential of this therapy. Further studies will focus on the combination of VSV, JAK inhibitors, and immune checkpoint inhibitors, as we previously showed the potential of combining a related OV with such immunotherapies.^42^

## Material and methods

### Cell lines and culture

All cell lines were isolated from stage III/IV epithelial ovarian cancer patients and have been previously described: high-grade serous carcinomas (HGSC) (OV2085, OV1946, TOV1946, OV866(2), TOV1369TR, OV1369(2), TOV2223G, TOV2929D, TOV2835EP, OV4453, OV4453(2), OV4453.3, TOV3041G, TOV2295, OV2295, OV2295(2), TO3291G, OV3291, OV4485, TOV3133D, TOV3133G, OV3133, OV3133(2), TOV3121D, TOV2881EP, TOV2978G, OV2978),^38^^,^^43^^,^^44^^,^^45^ endometrioid carcinoma (TOV112D),^46^ clear cell carcinoma (TOV21G, TOV3392D),^43^^,^^46^ mucinous carcinoma (TOV2414),^43^ and unspecified adenocarcinoma (OV90, OV3331).^43^^,^^46^ Relevant information such as the cancer types, as well as the age of the patient at the time of diagnosis and the stage of the cancer when the cells were isolated can be found in Table 1.

All ovarian cancer cell lines were cultured in OSE medium (316-030-CL, Wisent Inc, Saint-Bruno, Canada) supplemented with 10% fetal bovine serum (FBS) (098–150, Wisent Inc), 2.5 μg/mL amphotericin B (450-105-QL, Wisent Inc), and 50 μg/mL gentamicin (15750060, Gibco, Billings, MT). African green monkey kidney epithelial Vero cells (ATCC) were cultured in DMEM (11995073, Gibco) supplemented with 10% FBS (098–150, Wisent Inc). All cell lines were maintained at 37°C with 5% CO_2_.

### Virus imaging and viral assays

The VSV used in this study is VSVΔ51, an oncolytic mutant of the Indiana strain with a deletion of methionine 51 in its M protein.^7^ In this study, we used a variant that was engineered to encode the YFP.^47^

For virus quantification, plaque assays were performed as described previously.^48^ In brief, confluent monolayers of Vero cells were infected 1 h at 37°C with serial dilutions of samples containing virus. Agarose overlays were poured onto infected cells and plaques were counted the following day.

### Fluorescence imaging

VSVΔ51-YFP infection was assessed by live fluorescence imaging. High-resolution pictures were acquired with a ZOE fluorescent cell imager (Biorad, Hercules, CA) and screens and fluorescence quantifications were performed using an Ensight multimode plate reader (PerkinElmer, Waltham, MA) and its associated software Kaleido version 3.0.

### Viability assays

Coomassie blue stains were used as a readout of cancer cell death as previously described.^14^^,^^48^ Briefly, cells were fixed for 30 min at room temperature (RT) in a 1:3 acetic acid:methanol solution and then stained using a 0.1% Coomassie blue staining solution (in 1:3 acetic acid:methanol) for 30 min at RT. Plates were then washed with tap water and dried. For signal quantification, a solution of 1% sodium dodecyl sulfate (Bioshop, Burlington, Canada) was added to the wells for 1 h and the optic density at 595 nm was measured using an Ensight multimode plate reader (PerkinElmer) and its associated software Kaleido version 3.0.

### Mice experiments and xenograft models

*In vivo* experiments were performed in accordance with the CRCHUM’s institutional animal care committee. For subcutaneous (s.c.) tumors, 2 × 10^7^ tumor cells in 100 μL of Dulbecco’s PBS (14040141, Gibco) were mixed with 100 μL of Cultrex Basement Membrane Extract (3432-010-01, R&D Systems Inc, Minneapolis, MN), and 200 μL was injected into the left flank of NOD.Cg-Rag1^tm1Mom^ Il2rg^tm1Wjl^/SzJ mice.^49^ For *ex -vivo* experiments, tumors were collected when they reached a maximal volume of 1,500 mm³ ((width^2^ ∗ length)/2) and processed as detailed in the dedicated section.

For the efficacy study, mice were injected s.c. with tumor cells and treated 4 months later when tumors reached 90 mm^3^. Treatment on day 0 consisted in intraperitoneal (i.p.) injections of 200 μL PBS or ruxolitinib (7064/50, Biotechne, 60 mg/kg) 4 h prior to intratumoral injections of 100 μL of PBS or VSVΔ51-Luc (10e8 plaque-forming units [PFU]). Tumors were measured three times a week using a manual caliper (13196-070, VWR, Radnor, PA). Mice were euthanized when tumors were above 500 mm^3^, and tumors were harvested on day 53 post-virus treatment for the remaining mice.

### *Ex vivo* infections using micro-dissected xenografts

*Ex vivo* infections were performed into microfluidic devices, which have been described previously.^50^ Each device consists of two layers of poly-dimethyl siloxane molded and assembled to form four channels, each of which contains eight wells. Devices were flushed with 100% ethanol, 70% ethanol, and then incubated overnight with a pluronic F-108 solution (10 mg/mL in PBS, 542342, Sigma Aldrich, St Louis, MO) to prevent tissue adherence. The following day, devices were sanitized with 70% ethanol and then incubated with Hanks’ balanced salt solution (HBSS) (311-516-CL, Wisent Inc) supplemented with 0.6 μg/mL amphotericin B (Wisent Inc), 55 μg/mL gentamicin (Gibco).

Tumor samples were first sectioned into 1-mm slices using a McIlwain tissue chopper (Ted Pella Inc, Redding, CA). Microdissection was then performed on the tissue slices using a 500-μm tissue biopsy punch (PUN0500, Zivic instruments, Pittsburgh, PA). The samples were kept in HBSS (Wisent Inc) supplemented with 0.6 μg/mL amphotericin B (Wisent Inc), 55 μg/mL gentamicin (Gibco) and 10% FBS (Wisent Inc) and loaded into the microfluidic devices as described previously.^50^

The next day, the culture medium was replaced by either fresh culture media or media containing ruxolitinib (final concentration of 5 μM) (7064/50, Biotechne, Minneapolis, MN). Infection was performed 4 h later using a dose of 1.5 × 10^6^ PFU/mL of VSVΔ51-YFP. Twenty-four hours post-infection, supernatants from each channel were collected for virus quantification and tumor samples were fixed in formalin as previously described^50^ for further examination by IF.

### IF stains and image analysis

IF stains were performed as described previously.^51^ The panel used included cleaved caspase-3 (CC3) (rabbit anti-human caspase-3 [ab13847, Abcam, Cambridge, United-Kingdom]), VSV (mouse anti-VSV G [Sigma Aldrich, SAB4200695]), cytokeratins (CK) 8 and 18 (mouse anti-human CK8 (sc-6259, Santa Cruz Biotechnology, Dallas, TX), a mouse anti-human CK18 (MA5-14428, ThermoFisher Scientific, Waltham, MA) and DAPI to quantify apoptotic cells, infected cells, tumor cells and nuclei, respectively. Antigen retrieval was performed using a Benchmark XT autostainer (Ventana Medical System Roche, Oro Valley, AZ). Slides were incubated with anti-VSV G and anti-CC3 primary antibodies for 1 h at RT and washed with PBS. Slides were then blocked using the protein-block from Agilent (X0909, Agilent, Santa Clara, CA) for 20 min and incubated for 1 h with secondary antibodies diluted PBS, 1% BSA (goat anti-mouse Cyanine 5 [ThermoFisher Scientific, A31571)]and goat anti-rabbit TRITC [A-11010, Life Technologies, Carlsbad, CA]). Slides were then incubated overnight with a mouse-on-mouse blocking reagent (MKB-2213, Vector Laboratories, Burlingame, CA). The next day, the slides were incubated with anti-CK8 and anti-CK18 antibodies for 1 h, washed with PBS, and incubated with a goat anti-mouse AF488 antibody (A-11001, Life Technologies) for 40 min and DAPI (d-3571, ThermoFisher scientific) was added to the slides. The slides were then washed and incubated 15 min in a 0.1% sudan black solution (199664 diluted in ethanol 70%, Sigma Aldrich) and mounted with fluoromount aqueous mounting medium (F4680, Sigma Aldrich). Images were acquired using an Olympus optical microscope (BX61VSF, Olympus, Tokyo, Japan).

### Drugs and antibody treatments

Drugs used in this study: carboplatin, doxorubicin hydrochloride, cyclophosphamide hydrate, gemcitabine, olaparib, baricitinib (all from Cayman Chemical, Ann Arbor, MI: 13112-25, 15007-5, 13849-1, 11690-10, 10621-5, 16707-50), paclitaxel (HY-B0015, Med Chem Express, Monmouth Junction, NJ), ruxolitinib (7064/50, Biotechne), or fedratinib (HY-10409, Med Chem Express). Human IFN beta 1a (11410-2, PBL assay science, Piscataway, NJ) or a mixture of human type I IFN neutralizing antibodies (39000-1, PBL assay science) were also used at the indicated concentrations for some experiments.

In all cases, tumor cells were pre-treated with the drugs, cytokine, or antibodies for 4 h and then infected with VSVΔ51-YFP at the indicated MOIs.

### ELISAs

Culture supernatants were collected from infected cells and ELISAs were performed to measure IFN production. For IFNβ, the capture antibody (MAB8144-100 in PBS, BSA 1%, R&D Systems Inc) was incubated at 4°C O/N in 96-well EIA/RIA assay microplates (CLS3590, Corning Inc, Corning, NY). The plates were then washed with PBS-Tween 0.05% and blocked in 1% BSA in PBS at RT for 1 h. Samples were incubated for 2 h at RT in duplicate and recombinant human IFNβ was used as a standard (11410-2, PBL assay science). The plates were then washed with PBS-Tween 0.05%, incubated with the capture antibody (MAB8143-100, diluted in 1% BSA in PBS, R&D Systems Inc, which was previously biotinylated using the Biotinylation Kit - Fast, Type A, (ab201795, Abcam) as per the manufacturer’s protocol) for 2 h at RT. Streptavidin-HRP (DY998, 1% BSA in PBS, R&D Systems Inc) was then added for 20 min at RT, followed by a TMB/E substrate solution (ES022, Millipore Sigma, Burlington, MA) for 10 min and a stop solution (DY994, R&D Systems Inc). The absorbance was measured at 450 nm using an Ensight multimode plate reader (PerkinElmer) and its associated software Kaleido version 3.0. Human IFNα production was measured using a commercial ELISA kit (41110, PBL assay science) as per the manufacturer’s instructions.

### Statistical analyses

Statistical analyses were performed using Graphpad Prism software 9.5.1 (GraphPad, La Jolla, CA, USA). Unpaired multiple t tests were performed to compare the different groups. Results were considered significant when *p* values were inferior to 0.5 (∗*p* < 0.05, ∗∗*p* < 0.01, ∗∗∗*p* < 0.001, ∗∗∗∗*p* < 0.0001).

### Conclusion

Oncolytic VSV effectively infects human ovarian cancer cell lines and xenografts and induces cell death, therefore demonstrating its potential as a therapy against the disease. We show that combinations with JAK1/2 inhibitors further improve therapy and allow for the killing of VSV-resistant ovarian cancer models. Altogether, our study demonstrates the potential of VSV and OV therapy against ovarian cancer.

## Data and code availability

Cell lines are a part of the OvCAN collection and are available to the Canadian research community for pre-clinical studies upon request.

## Supplemental information

Document S1. Figures S1–S7

Document S2. Article plus supplemental information
