# Supplementary material for: Oncolytic vesicular stomatitis virus alone or in combination with JAK inhibitors is effective against ovarian cancer
Source: Mol Ther Oncol. 2024 Jun 8;32(3):200826. doi: 10.1016/j.omton.2024.200826 (PMC11246050; doi:10.1016/j.omton.2024.200826)
Supplement: Document S1. Figures S1–S7 [file mmc1.pdf]

OMTON, Volume *32*

Supplemental information

Oncolytic vesicular stomatitis virus alone or in combination with JAK inhibitors is effective against ovarian cancer

Karen Geoffroy, Victor Mullins-Dansereau, Kim Leclerc-Desaulniers, Mélissa Viens, and Marie-Claude Bourgeois-Daigneault

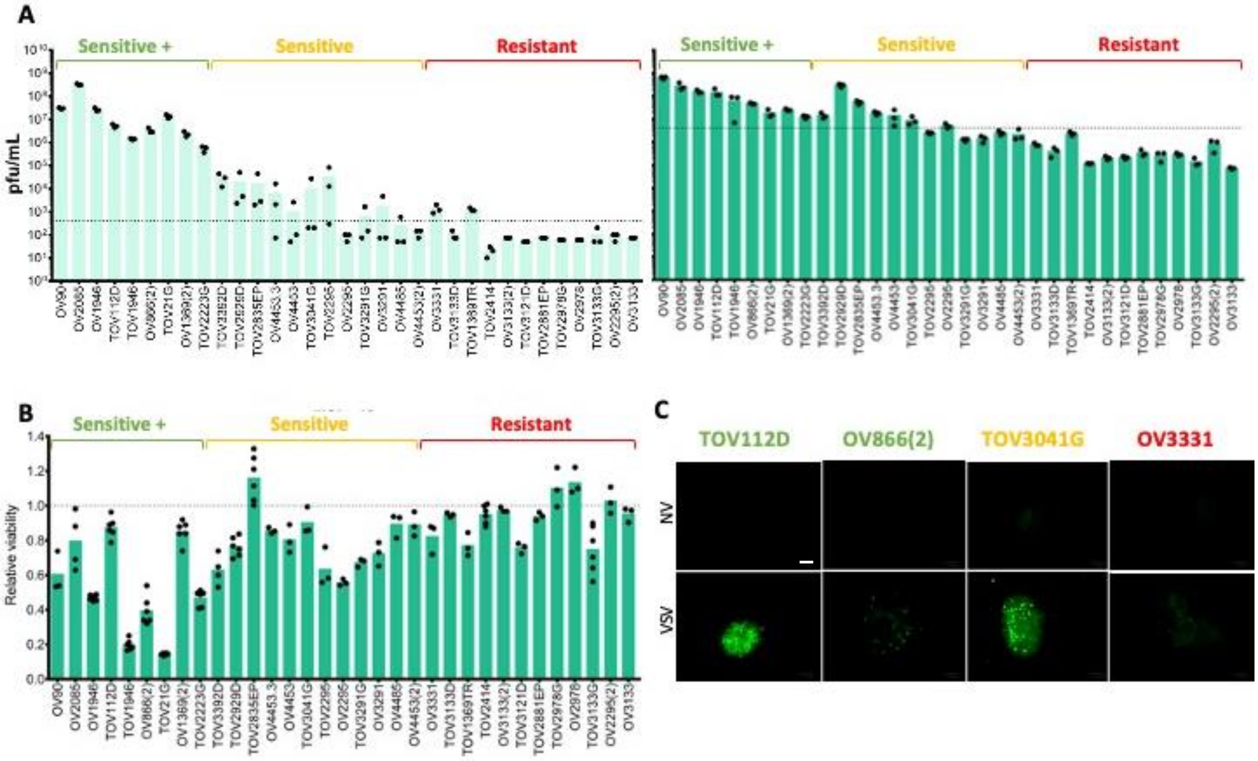

Figure S1. Ovarian cancer cell lines have heterogenous sensitivities to VSV infection.

(A) Viral outputs from supernatants from ovarian cancer cells infected for 24h at MOIs of 0.001 (left panel) or 10 (right panel) were measured by plaque assay. The dotted line indicates virus inputs (n=3). **(B)** Cellular viability from (A, right panel) as determined by coomassie blue staining and relative to non-infected cells (n≥3). **(C)** Fluorescence imaging pictures of micro-dissected xenografts 24h post-infection with VSVΔ51-YFP (1.5x10^7 PFU/mL for TOV112D or 1.5x10^8 PFU/mL for OV866(2), TOV3041G and OV3331). Scale-bar = 100 µm.

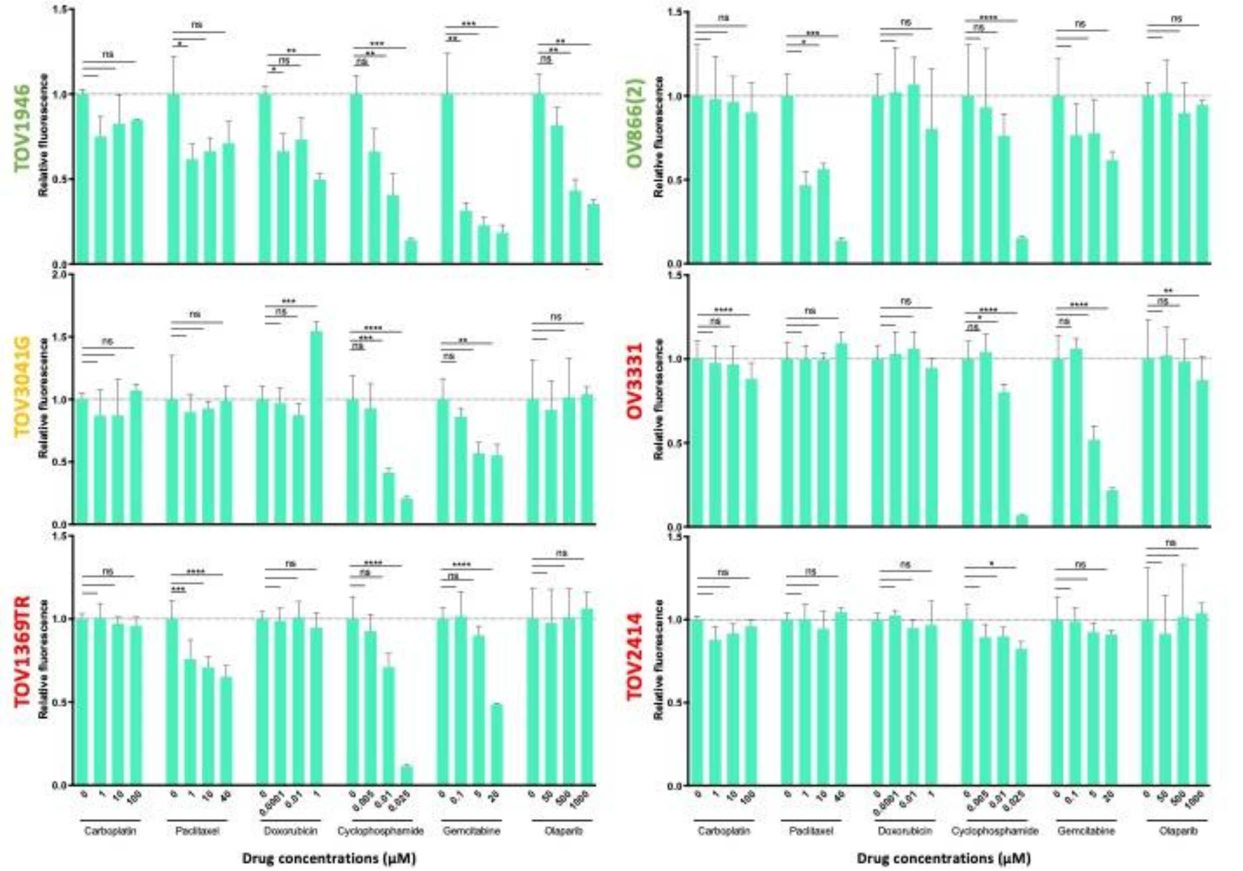

Figure S2. Combinations of VSV with various anticancer drugs do not enhance VSV.

Relative fluorescence signal (infection) of ovarian cancer cell lines infected with VSVΔ51-YFP 4h post-carboplatin, -paclitaxel, -doxorubicin, -cyclophosphamide, -gemcitabine or -olaparib treatment at the indicated concentrations compared to no drug controls. MOIs used: 0.1 for TOV1946 and OV866(2), 10 for TOV3041G and OV3331 or 100 for TOV1369TR and TOV2414. The fluorescence was measured 24h post-infection using an Ensight multimode plate reader. Two-way ANOVA test, ns: $p > 0.05$, *: $p \leq 0.05$, **: $p \leq 0.01$, ***: $p \leq 0.001$, ****: $p \leq 0.0001$.

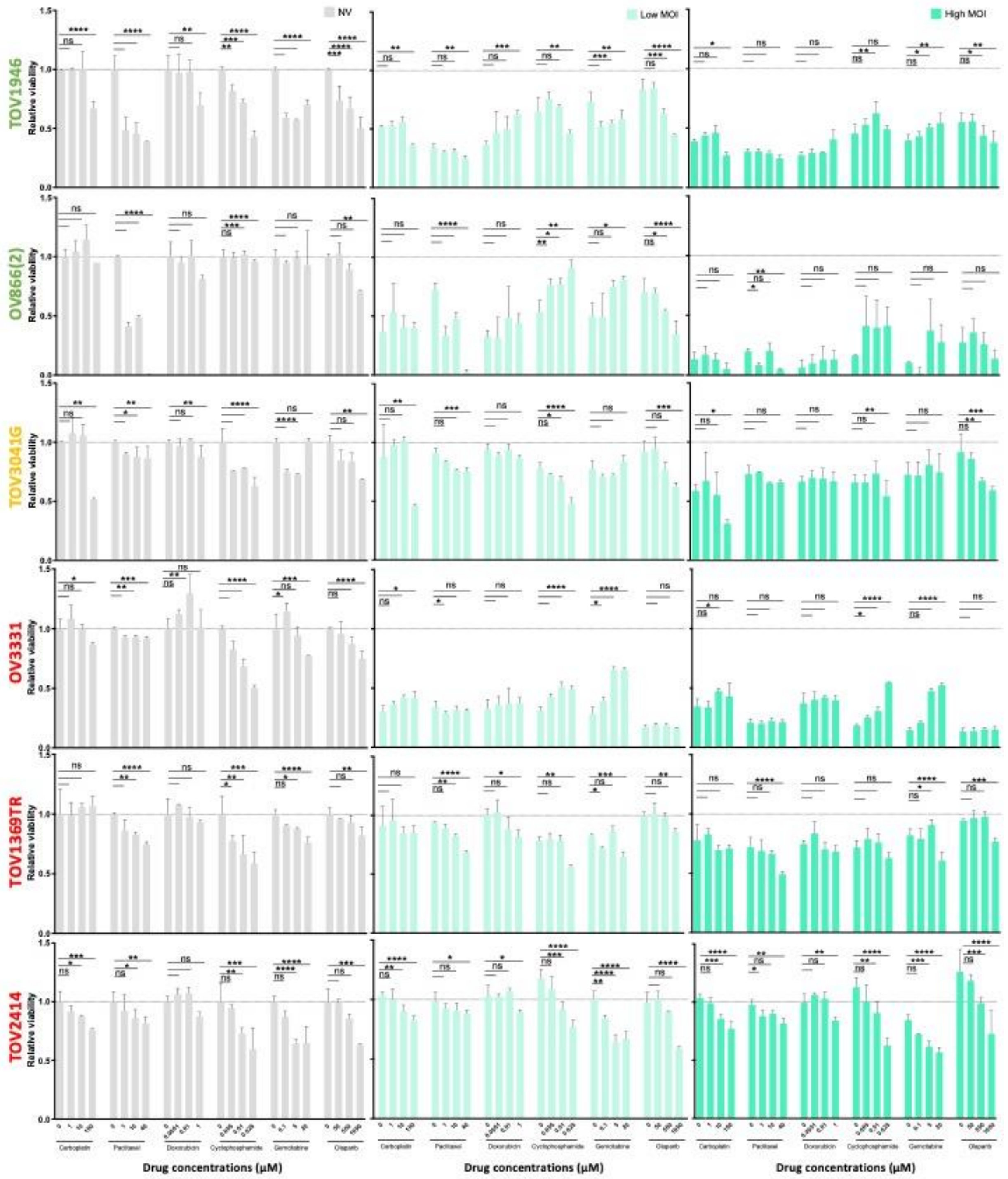

Figure S3. Combinations of VSV with anticancer drugs do not improve cancer cell death.

Cell viability, as assessed by coomassie blue staining, of samples from Figs. 2 and S2. Dotted lines represent the viability of the corresponding control condition (without drug). Two-way ANOVA test, ns: $p > 0.05$, *: $p \leq 0.05$, **: $p \leq 0.01$, ***: $p \leq 0.001$, ****: $p \leq 0.0001$.

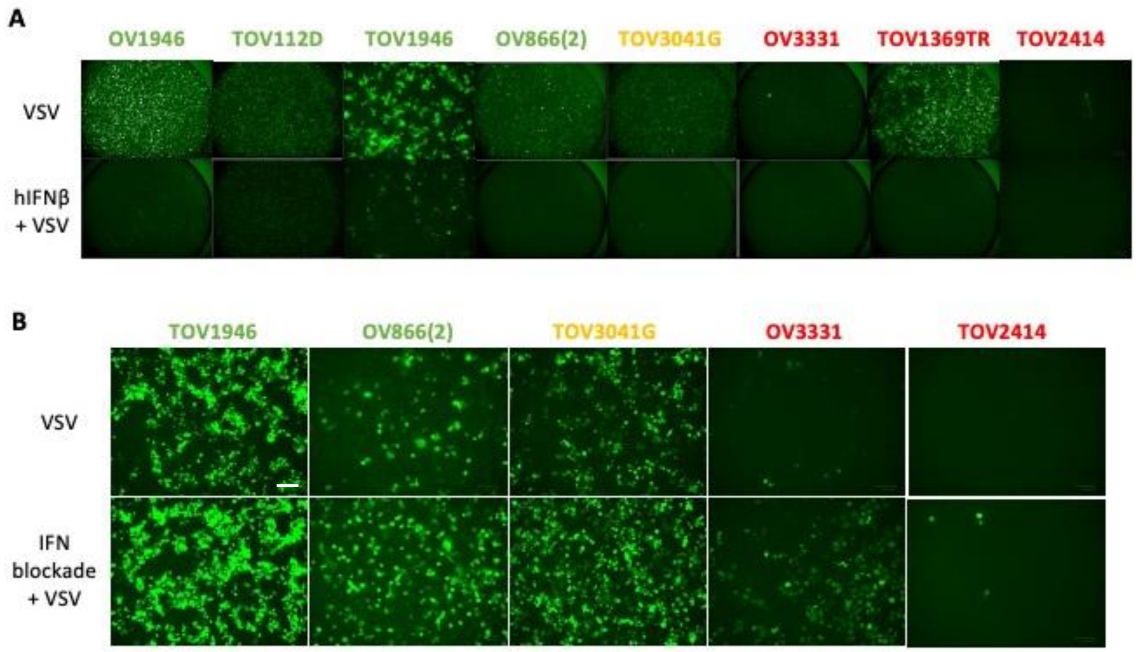

Figure S4. IFN responsiveness of ovarian cancer cells.

Representative fluorescence imaging pictures of ovarian cancer cell lines with or without **(A)** IFNβ or **(B)** type I IFN neutralizing antibodies treatment for 4h prior to infection with VSVΔ51-YFP for 24h at an MOI of 10. Scale-bar = 100µm.

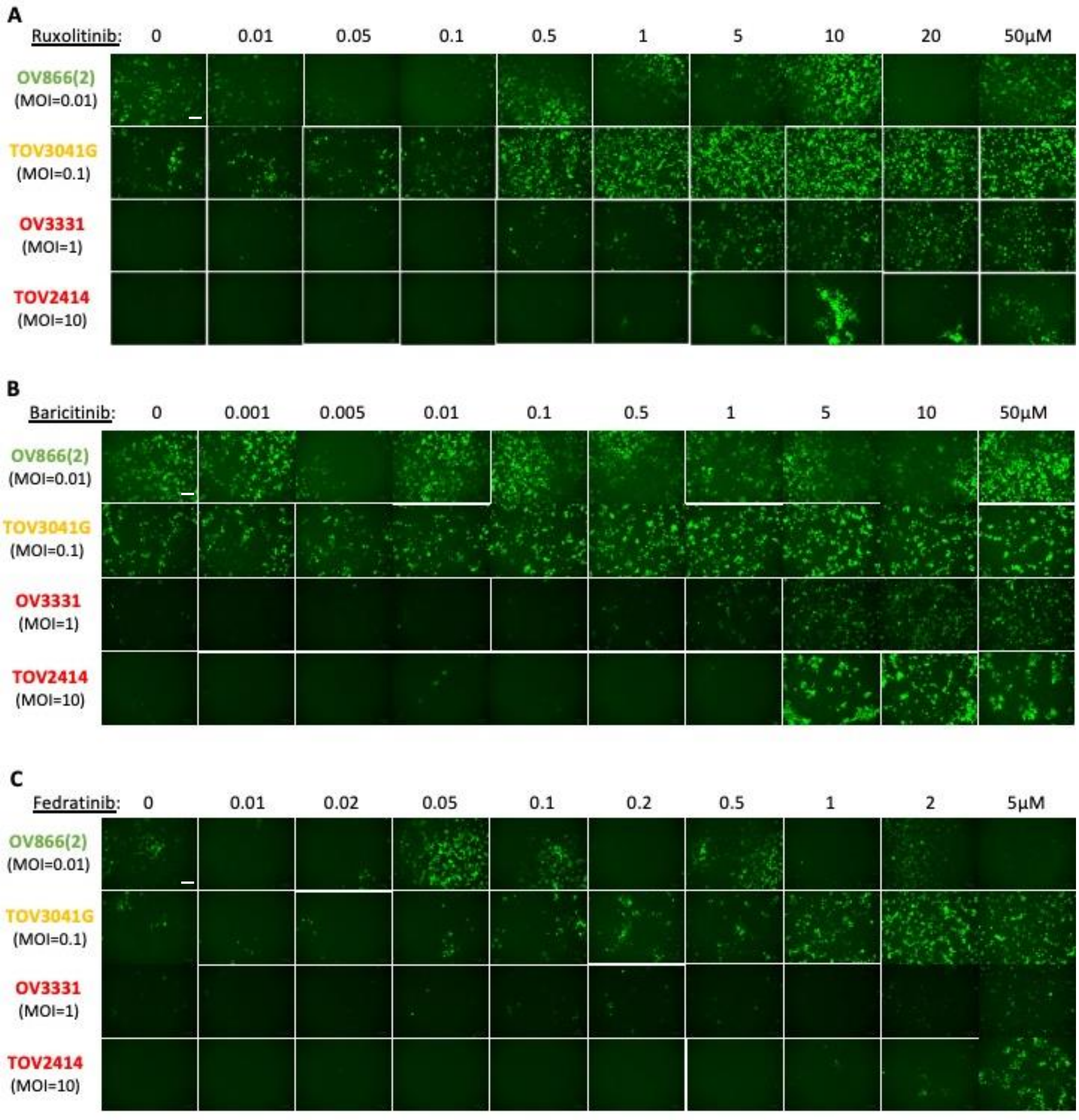

Figure S5. Combinations of VSV and JAK inhibitors increase viral replication.

Fluorescence imaging pictures of OV866(2), TOV3041G, OV3331 and TOV2414 cells treated with **(A)** ruxolitinib, **(B)** baricitinib or **(C)** fedratinib at the indicated concentrations and infected 4h later with VSVΔ51-YFP at the indicated MOIs. Pictures were taken 24h post-infection. Scale-bar = 100µm.

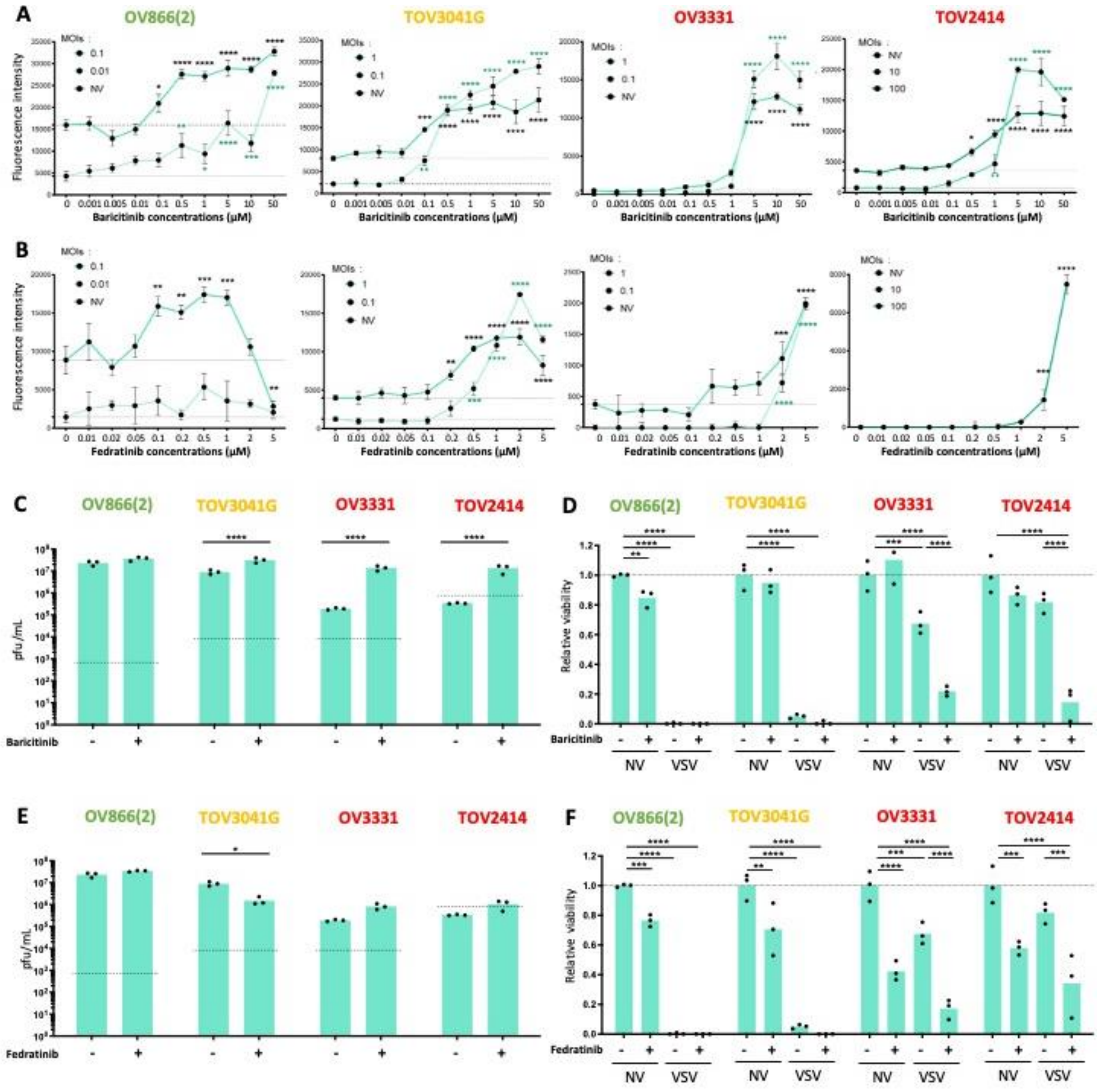

Figure S6. Combinations of VSV and JAK inhibitors increase viral replication and cell death.

Fluorescent signal from cells treated with various concentrations of **(A)** baricitinib or **(B)** fedratinib and infected 4h later with VSVΔ51-YFP for 24h was quantified. Viral outputs from culture supernatants from cells pre-treated with **(C)** baricitinib (10µM) or **(E)** fedratinib (2µM or 1µM for OV866(2) cells) and infected for 48h with VSVΔ51-YFP (MOIs used: 0.01 for OV866(2), 0.1 for TOV3041G and OV3331, and 10 for TOV2414) were measured by plaque assays and **(D)** cell viability was measured by Coomassie blue staining from (C) and **(F)** from conditions as in (E). Two-way ANOVA test (n=3), *: p ≤ 0.05, **: p ≤ 0.01, ***: p ≤ 0.001, ****: p ≤ 0.0001.

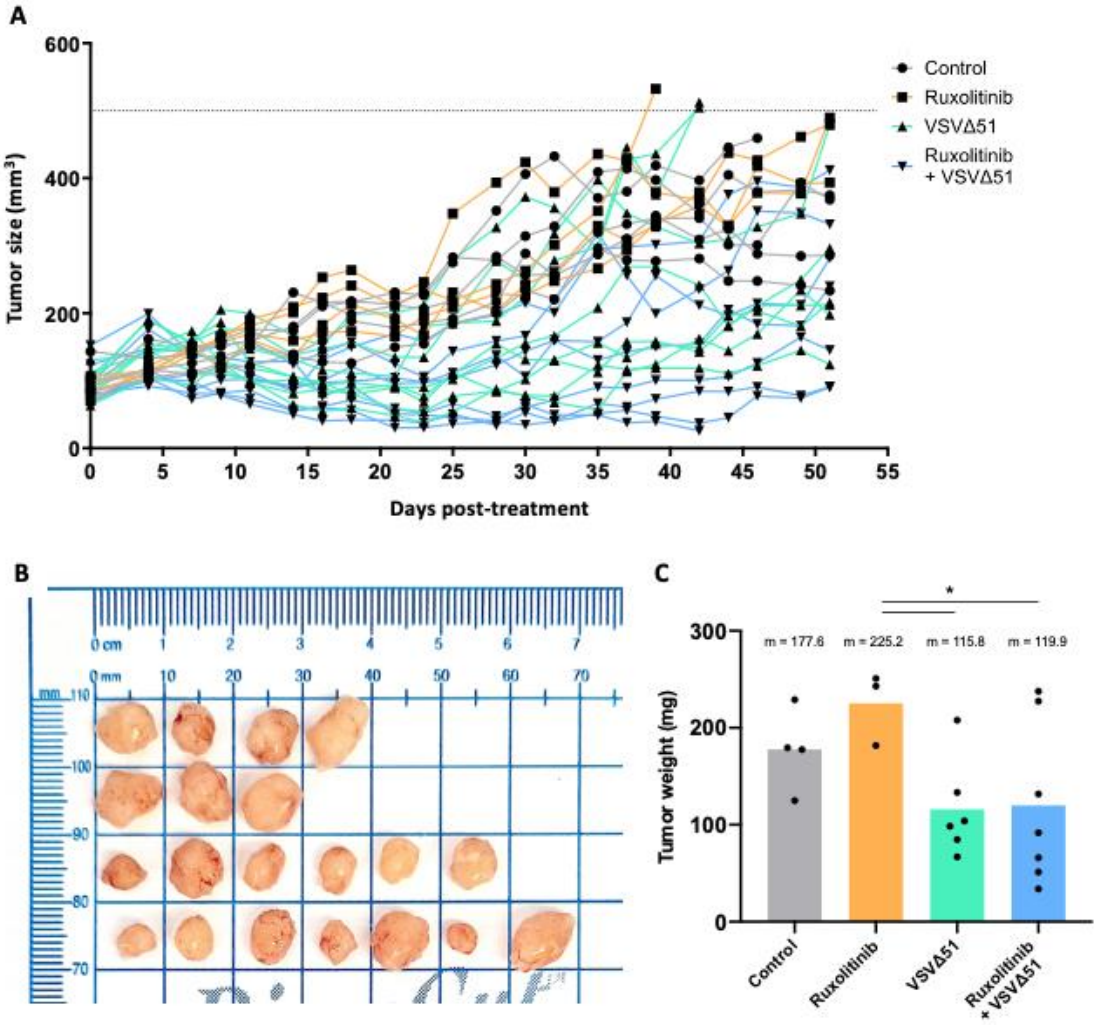

Figure S7. Tumors from mice treated with VSV alone or in combination had smaller tumors.

(A) Tumors were measured from day 4 to 53 post-treatment and **(B)** were harvested on day 53 to compare size in each group (line 1: control, line 2: ruxolitinib, line 3: VSVΔ51, line 4: Ruxolitinib + VSVΔ51) and **(B)** tumors were weighted. M = mean. Multiple t-test, *: $p \leq 0.05$.
